# What Is Distinctive About Autism Arising Following Severe Institutional Deprivation? A Direct Comparison With a Community Sample of Early Diagnosed Autistic People

**DOI:** 10.1002/aur.70026

**Published:** 2025-03-26

**Authors:** Maria Rodriguez‐Perez, Susie Chandler, Mark Kennedy, Tony Charman, Emily Simonoff, Edmund Sonuga‐Barke

**Affiliations:** ^1^ Department of Child and Adolescent Psychiatry, Institute of Psychiatry, Psychology & Neuroscience King's College London London UK; ^2^ Department of Psychology, Institute of Psychiatry, Psychology & Neuroscience King's College London London UK; ^3^ Department of Child & Adolescent Psychiatry Aarhus University Aarhus Denmark; ^4^ Department of Psychology University of Hong Kong Hong Kong Hong Kong

**Keywords:** autism, early adversity, institutional deprivation, longitudinal, Romanian adoptees

## Abstract

In the English and Romanian Adoptees study, a substantial proportion of adoptees who suffered extended severe deprivation (26 of 101) displayed autistic characteristics termed quasi‐autism (QA). Here we directly compare this group with a community sample of early diagnosed autistic individuals (community autism; CA). First, we characterized the QA autism symptom profile (61.5% females) by calculating which of the 32 Social Communication Questionnaire (SCQ) items were statistically more common in the QA group than in a control group of 52 non‐deprived UK adoptees (UK Control, 34.6% females) at ages 11, 15, and/or 23 years of age. The latent structure of these QA‐characteristic items was explored using confirmatory factor analyses. Second, we compared the QA symptom profiles with CA profiles using a sample from the QUEST study (Salazar et al. 2015). To do this, we identified two QUEST groups, one aged 11 years on average (*n* = 21) and one aged 15 years (*n* = 24). The former were compared to ERA SCQ scores at age 11, and the latter at age 15. Nineteen SCQ items were statistically significantly more common in the QA group than in the ERA UK control group at ages 11 and 15. Ten differences persisted into adulthood. These QA‐characteristic items ranged across and mapped onto all three standard SCQ domains (social reciprocity, communication, repetitive and stereotyped behaviors). The age 11 CA group scored higher than QA at 11 years across each subscale when all items were considered. However, when only QA‐characteristic items were included, only scores for the Repetitive and Stereotyped subscale differentiated QA and CA. When the age 15 comparison was made, no differences were found between CA and QA subscales. QA and CA were associated with similar levels of emotional and conduct problems and overactivity/inattention levels. QA shared many features with CA. QA difficulties extended across all autism domains and were associated to a similar degree with emotional and behavioral problems. However, there were some distinctive elements. Compared to the classic autism profile, the communication domain mainly comprised persistent abnormalities of linguistic expression. In contrast, social reciprocity problems were diffuse, less severe, and declined over time. QA‐characteristic repetitive and stereotyped behaviors are broadly expressed and endure into adulthood.

**Summary**

- Autism arising following institutional deprivation (i.e., QA) shares many features with community autism (i.e., CA), with difficulties extending across all domains and being associated to a similar degree with emotional and behavioral problems when compared to CA.
- However, compared to the classic autism profiles, the communication domain mainly comprises persistent abnormalities of linguistic expression.
- In contrast, social reciprocity problems are diffuse, less severe, and decline over time.
- Finally, repetitive and stereotyped behaviors are broadly expressed and endure into adulthood.

Autism is a neurodevelopmental condition that is clinically characterized by communication and social interaction difficulties, as well as the presence of restricted, repetitive, and stereotyped behaviors, interests, or activities, and sensory anomalies (American Psychiatric Association 2013). The condition typically has strong genetic underpinnings, as suggested by twin and molecular studies (Lord et al. 2020). Environmental factors also appear to play a role (Gardener et al. 2011; Hallmayer et al. 2011). Environmental risks linked to autism can generally be divided into three groups: (i) preconception factors, such as parental age (Gardener et al. 2009) and maternal obesity (Lei et al. 2019); (ii) prenatal factors, such as exposure to viral or bacterial infections, toxins, and maternal stress (Arndt et al. 2005; Beversdorf et al. 2005; Karr et al. 2007; Patterson 2009); and (iii) perinatal factors, like prematurity, pregnancy, and birth complications (Badawi et al. 2006; Crump et al. 2021; Gardener et al. 2011; Hisle‐Gorman et al. 2018).

There is little evidence that postnatal environmental factors increase risk. Breaking with this general pattern, Rutter et al. (1999), as part of the English and Romanian Adoptees (ERA) study, found that individuals exposed to more than 6 months of extreme global deprivation early in life in the Romanian institutions at the end of the 1980s were at a strikingly elevated risk of displaying marked autism features. Twenty children were identified at age 4/6 (Rutter et al. 1999) as meeting the clinical threshold on the *Autism Diagnostic Interview* (ADI; Lord et al. 1994) and the *Autism Diagnosis Observation Scale* (ADOS, Gotham et al. 2006). A further 8 children were identified at age 11/12 (Rutter et al. 2007) as meeting a cutoff of over 14 on the total score of the *Social Communication Questionnaire* (SCQ; see Rutter et al. 2007 for rationale).

When they were aged 4 years, Rutter et al. (1999) compared the autistic features displayed by these individuals with more typical autistic presentations in a small non‐deprived clinical sample. The differences observed included greater communicative flexibility, an uncommon degree of social approach, and an unusually high degree of odd preoccupations (e.g., watches, wires, new £10 notes, light switches, etc.), and sensory interests (i.e., smells or tactile sensations) in the deprived group. There also seemed to be a greater remission of symptoms between ages 4 and 6 years in this group, as well as a higher proportion of females. Based on these differences, Rutter et al. (1999) termed this deprivation‐related autism symptom pattern “*quasi‐autism*” (QA). In the ERA study, QA was expressed as part of a broader pattern of deprivation‐specific neurodevelopmental problems, also including attention‐deficit/hyperactivity disorder (ADHD) (Kennedy et al. 2016), disinhibited social engagement (DSE; initially described as disinhibited attachment) (Kennedy et al. 2017), and cognitive impairment (i.e., IQ < 80; Rutter et al. 2010). Other studies have also found elevated levels of autism symptoms in institutionally deprived groups (Federici 1998; Hoksbergen et al. 2005; Levin et al. 2015) and those experiencing disrupted care and/or maltreatment (Green et al. 2016; Wolstencroft et al. 2023). Although the reasons for the elevated rates of autism in children exposed to deprivation and neglect still need to be determined, these findings support the notion that exposure to severe neglect and deprivation, and perhaps other forms of maltreatment, may determine autism symptom trajectories in some individuals.

Recently, we reported on the child‐to‐adult developmental trajectories of autism and associated problems, and young adult outcomes in this QA group (Rodriguez‐Perez et al. 2023). Key findings included the following. First, following a degree of early catch‐up (Rutter et al. 1999), there was a strong continuity of autism features into adulthood despite the adoptees having been raised in well‐functioning families for over 20 years after adoption from the institutions. Second, in adulthood, the QA group continued to manifest symptoms across all three autism domains (i.e., communication, social reciprocity, and repetitive and stereotyped behaviors), although there were subtle differences in developmental trajectories between domains, with communication problems apparently worsening to some extent and repetitive and stereotyped behaviors, although persistent, improving to a degree. Third, individuals in the QA group also displayed the other deprivation‐specific problems observed in the ERA cohort (i.e., symptoms of ADHD, DSE, and cognitive impairment). Fourth, individuals in the QA group exhibited a complex set of challenges in adulthood, as highlighted by the high levels of mental health problems and functional impairment in this group (Edwards et al. 2023). Lastly, despite all their challenges, individuals in the QA group displayed good relationships with parents and a sense of self‐worth and life satisfaction. Overall, we concluded that individuals in the QA group observed across the period from early childhood to adulthood seemed to share many elements of the symptom profile, co‐occurring functional impairment, and mental health problems with clinically diagnosed community‐based autism. However, this conclusion was based on an indirect comparison with data in other published papers rather than a direct comparison with a cohort of young people with autism. In addition, findings were reported in terms of aggregated symptoms by domain (i.e., communication, social reciprocal interactions, and repetitive and stereotyped behaviors) and, therefore, we were unable to explore in a more granular way the symptomatic profile of the QA group.

In the current study, we extend this analysis to explore these issues in a more direct and detailed way. There are two stages to the analysis. In stage one, our goal is to provide a detailed characterization of the autism profile displayed by the QA group by identifying which autism symptoms are significantly more likely to be present in the QA group (*n* = 26 Romanian adoptees) than in a group of non‐deprived UK adoptee controls, also part of the ERA study, at three different ages: 11, 15, and 23 years. Subsequently, we used confirmatory factor analysis in the ERA sample that had experienced extended deprivation (i.e., > 6 months of exposure to deprivation) to test whether these QA‐group‐differentiating items statistically mapped onto the standard domains as per either the DSM‐IV (APA 1994) or DSM‐5 (APA 2013). In stage two of the analysis, we compared the autism profile of the QA group and levels of associated emotional and behavioral problems with a community sample of early diagnosed autistic individuals (hereafter community autism: CA) from the QUEST study (Carter Leno et al. 2022; Hollocks et al. 2023; Salazar et al. 2015). The QUEST cohort was chosen as a comparator because CA individuals were not exposed to early global institutional deprivation and had received a clinical diagnosis of autism in early childhood. Furthermore, the timing of adolescent CA assessments overlapped with two of the follow‐up periods in ERA, that is, age 11 and age 15. To take advantage of this and to account for the overall differences in the age of assessment, two CA groups were selected from the total QUEST sample. One group (CA‐11) consisted of 21 eleven‐year‐olds, to map the earlier ERA adolescent follow‐up, and the other (CA‐15) consisted of 24 fifteen‐year‐olds, to match the later follow‐up.

Our research questions were as follows:

1. What is the autism profile of the QA group at ages 11, 15, and 23 years?
2. Do the QA group's autism symptoms cluster according to the standard autism domains?
3. How do autism symptoms in the QA and CA groups differ during adolescence (i.e., age 11 and 15)?
4. Do QA and CA groups show similar patterns of co‐occurring behavioral and emotional difficulties during adolescence?

## 1 Methods

### 1.1 Participants

The sample in the current study consisted of individuals from the ERA and the QUEST studies.

#### 1.1.1 ERA Study Samples

The full ERA sample is described in detail in earlier papers (e.g., Sonuga‐Barke et al. 2017). In brief, the ERA study is a longitudinal, multi‐method investigation of the development of individuals adopted into the UK from Romanian orphanages. The full sample consists of 165 Romanian adoptees and 52 comparison UK adoptees with no history of institutional deprivation, and their families, who initially took part in the study in the mid‐1990s. Romanian adoptees typically entered the institutions in the first few months of life and experienced between 1 and 43 months of extreme early global deprivation. The study was approved by the ethics committees at King's College, London, UK (5447) and the University of Southampton, UK (14308). At each wave, all participants (adoptees and their parents) gave written informed consent or verbal assent, as developmentally appropriate. The ERA study retention rates were high up to age 15 years (data available for around 90% of the sample) but decreased somewhat into adulthood (data available for around 75% of the sample). There was no evidence of selective attrition based on a comparison between those dropping out prior to the age 11 wave and those who remained in the study (Sonuga‐Barke et al. 2017).

For the current paper, two groups of individuals were selected from the ERA sample. The first group included 101 individuals (59 females, 59.6%) who had experienced more than 6 months of deprivation. The 6‐month threshold has been used in previous ERA studies (e.g., Sonuga‐Barke et al. 2017) as demarcating a group at significantly increased risk for elevated levels of persistent neuro‐developmental problems. Twenty‐eight of this group were historically included in the *QA group*, consisting of 20 individuals who were identified with marked autistic features based on clinical assessment conducted at both/either 4/6 years of age (ADI; Lord et al. 1994; ADOS; Gotham et al. 2006), and a further 8 adoptees who met screening cut‐offs on the SCQ only (i.e., SCQ total score  >  14 current form; see Rutter et al. 2007 for rationale) at age 11/12. All had been exposed to more than 6 months of institutional deprivation. In the current study, two children were excluded from this group as they had been exposed to further biological risk factors (i.e., foetal alcohol syndrome) in addition to institutional deprivation, bringing the total QA participants to 26 (16 females, 61.5%) (Table 1). There were no significant differences in terms of autism symptoms between the 20 individuals who met the ADOS/ADI criteria and the 6 who just met the SCQ thresholds at age 11/12, 15, and young adulthood (see Appendix II, Table I). The second ERA group was the non‐deprived control group, which consisted of 52 UK adoptees who had no history of institutional deprivation (i.e., hereafter *UK Control*). One individual from this group had a childhood diagnosis of autism. ERA assessments were made at ages 4 and/or 6 years and then 11, 15 years, and young adulthood (average 23.5 years). For the purposes of the current analysis, data at ages 11, 15, and 23 years is used.

**TABLE 1:** Background characteristics of ERA and QUEST group members.

	ERA		QUEST		Main effect	Post hoc comparison
	QA (*n* = 26)	UK (*n* = 52)	CA‐11 (*n* = 21)	CA‐15 (*n* = 24)		
Sex (females, %)	16 (61.5%)	18 (34.6%)	5 (11.6%)	4 (9.3%)	***X*** ^**2**^ **(3) = 12.76**, ***p* = 0.005**	QA>UK > CA‐11 & CA‐15
Length of deprivation, months (mean, SD)	18.50 (10.51)	0.00 (0.00)	0.00 (0.00)	0.00 (0.00)	***F* (2**, **121) = 114.10**, ***p* < 0.001**	QA>CA‐11 & CA‐15 & UK
Age 11 (mean, SD)	11.20 (0.24)	11.08 (0.24)	11.67 (0.22)	—	***F* (2**,**94) = 46.38**, ***p* < 0.001**	CA‐11 > QA>UK
Age 15 (mean, SD)	15.63 (0.38)	15.25 (0.20)	—	15.32 (0.25)	***F* (2**,**87) = 15.046**, ***p* < 0.001**	QA>CA‐15 & UK
Parental level of education						
Mother (%, undergraduate degree)	14 (26.9%)	7 (26.9%)	11 (52.4%)	7 (50%)	*X* ^2^ (3) = 4.961, *p* = 0.175	—
Father (%, undergraduate degree)	19 (38.8%)	12 (52.2%)	7 (50%)	7 (46.7%)	*X* ^2^ (3) = 1.398, *p* = 0.706	—

*Note*: Figures in **bold** show groups that are significantly different (i.e., *p* < 0.05).

Abbreviations: CA, Community Autism; QA, Quasi‐Autism; SD, Standard Deviation.

#### 1.1.2 QUEST Sample—Early Identified Community Autism

The QUEST study (see Salazar et al. 2015, for details) is a community sample of 277 individuals born between 2001 and 2004 (aged 4–8 years at time of recruitment) with a childhood diagnosis of autism at entry to the study (for details of diagnostic measures see Salazar et al. 2015) from two London health districts (i.e., Lewisham and Bromley), originally assessed at age 4–9 (wave 1), then age 11–15 (wave 2) (Carter Leno et al. 2022), and most recently at age 13–17 (wave 3) (Hollocks et al. 2023). All participating families gave their written informed consent, and the study was approved by Guy's Hospital Research Ethics Committee (08/H0804/37) at wave 1 and Camden and King's Cross Ethics SubCommittee at wave 2 (14/LO/2098) and wave 3 (17/LO/0397). All participants had a community clinical diagnosis of autism by age 7 years, and a portion of them (i.e., 41) had their diagnosis reconfirmed at age 11–15 years with the ADOS and ADI. All participants were above threshold on either or both the ADOS and the ADI at age 11–15 years. To the best of our knowledge, QUEST children were not exposed to institutional deprivation. Five children had lived in foster care for some of their lives, and one child had been adopted. QUEST followed up 76% of the original number of families at age 11–15 follow‐up, and 77% at the 13–17 assessment point. There was no evidence of selective attrition when a comparison between those dropping out prior to the 13–17 follow‐up and those who remained in the study was performed (Hollocks et al. 2023). For the purposes of the current analysis, two groups, the CA‐11 and CA‐15 (21 eleven‐year‐old (5 females, 11.6%) and 24 fifteen‐year‐old (4 females, 9.3%), respectively), were selected and compared to the ERA sample at ages 11 to 15 as described above (Table 1). There were no significant differences in terms of autism symptoms between those QUEST cases selected and those excluded in our analyses (Appendix III, Table III).

### 1.2 Measures

#### 1.2.1 Background Characteristics

For ERA Romanian adoptees' sex, date of birth, and duration of institutional deprivation was obtained from Romanian records taken at the time of entry into the United Kingdom. Equivalent data were collected for the ERA UK Control group at either enrolment in the study (i.e., sex and date of birth) or during each assessment point (i.e., exact age). For the QUEST participants' sex data was collected through parental questionnaires at age 4–9 years, whereas exact age was obtained at each assessment point by the same means. Parental level of education was obtained from parental questionnaires at enrollment in the study for all groups (age 4/6 for QA and UK Control groups, and 4–9 for CA).

#### 1.2.2 Autism Symptoms

An adapted version of the *current* form of the Social Communication Questionnaire (SCQ; Rutter et al. 2003), a clinically validated parent‐report screening test for autism symptomatology that maps onto the three DSM‐IV (APA 1994) diagnostic criteria, was used to assess autism symptoms across the three core domains—social reciprocity, communication, and repetitive and stereotyped behaviors at ages 11, 15, and 23 years for the ERA groups, and at the 11–15 year assessment point in QUEST. The SCQ has high internal consistency (Cronbach's alpha = 0.87; Rutter et al. 2003), and good discriminative validity when distinguishing between children with autism and non‐autistic children at all IQ levels, particularly when administered in children 4 years of age or older (Berument et al. 1999). The original SCQ consists of 40 items. However, in the ERA study, five items were dropped from age 11 as these were deemed inappropriate for assessment of a sample who were verbal. In the current study, we also excluded an additional three items as these do not constitute part of any subscale (i.e., communication, social reciprocity, and repetitive and stereotyped behaviors) (see Appendix I for details). The 32 selected items were rated by parents using a binary code (0 for “No” or 1 for “Yes”). Reverse scoring (i.e., recoding responses so that the numerical scoring scales runs in the opposite direction) was performed where relevant, so that a score of 1 would always reflect a positive symptom endorsement.

#### 1.2.3 Inattention and Overactivity Symptoms & Emotional and Conduct Problems

Inattention and overactivity (i.e., hyperactivity, sustained attention, impulsivity and distractibility), and emotional (i.e., depressed mood, worry, and social anxiety) and conduct problems (i.e., dishonesty, fighting and defiance) were assessed using the same items in the ERA and QUEST groups. For the ERA groups (i.e., QA and UK Control), these were collected using the Revised Rutter Scale (Elander and Rutter 1996) at age 11 and the Strengths and Difficulties Questionnaire (SDQ; Goodman 1997) at age 15. In QUEST, they were measured using the SDQ in both age groups. A symptom was deemed endorsed when a rating of 2 (“certainly applies”) was made (0–2 scales).

#### 1.2.4 IQ

For the ERA groups, cognitive function was assessed using the short form (i.e., block design, objects assembly, vocabulary, and similarities) of the Wechsler Intelligence Scale for Children (WISC, Wechsler 1974) at ages 11 and 15. For QUEST participants, verbal IQ was estimated using the Wechsler Abbreviated Scale of Intelligence (WASI‐II, Wechsler, 2011) at ages 11–15 and 13–17. IQ values were only collected for a minority of the selected QUEST participants (11 out of 21 eleven‐year‐olds and 6 out of 24 fifteen‐year‐olds). For QUEST, the Adaptive Behavior Assessment System‐II (ABAS‐II; Harrison and Oakland 2003) was also collected at wave 2 (age 11–15) for all participants. The ABAS asks parents/carers for information across nine skill domains (i.e., Communication, Community use, Functional Academics, Home‐living, Health/Safety, Leisure, Self‐care, Self‐direction, and Social). Domain scores are used to generate a general adaptive composite.

### 1.3 Statistical Analysis

#### 1.3.1 What Is the Autism Profile of the QA Group at Ages 11, 15, and 23 Years/Young Adulthood?

We compared the level of endorsement of each SCQ item in the QA and the UK Control group using chi‐square (*χ* ^2^) and Fisher's exact tests at 11, 15, and 23 years of age. Items significantly more common in the QA group versus the UK were referred to as “*QA‐characteristic*”.

#### 1.3.2 Do the QA Group's Autism Symptoms Cluster According to the Standard Autism Domains?

We conducted a series of Confirmatory Factor Analyses (CFA) on the SCQ items that we identified as QA‐characteristic. The ERA group with over 6 months of exposure to institutional deprivation (*n* = 101) was used for this analysis. Weighted least squares mean (WLSM), variance adjusted estimator, and oblique factors were applied. The over 6 months group was used at this point as they had experienced extended deprivation, and we were interested in the structure of deprivation‐related autism characteristics as a trait expressed beyond the QA group. Our prior analyses demonstrated that individuals exposed to over 6 months of deprivation but not identified as being part of the QA group had elevated scores on the SCQ, though in the sub‐clinical range (Rodriguez‐Perez et al. 2023). We tested three models: (a) Three‐factor model as per DSM‐IV (APA 1994); social reciprocity, communication and repetitive and stereotyped behaviors; (b) Two‐factor model as per DSM‐5 (APA 2013; socio‐communication difficulties, and repetitive and stereotyped behaviors), and (c) One‐factor model (See Appendix III for details). Model fit was evaluated using four descriptive fit indices: the standardized root‐mean‐square residual (SRMR), the root‐mean‐square error of approximation (RMSEA) with 90% confidence intervals, the comparative fit index (CFI), and the Tucker‐Lewis index (TLI). These indices have demonstrated robust performance under various data and model misspecification conditions (Hu and Bentler 1998; McDonald and Marsh 1990; Satorra and Bentler 1994). The criteria used to indicate good fit was as follows: a non‐significant *χ* ^2^ test (i.e., *p* >  0.05); SRMR ≤ 0.08; RMSEA ≤ 0.05; CFI ≥ 0.95; and TLI ≥ 0.95.

#### 1.3.3 How Do Autism Symptoms in the QA and CA Groups Differ During Adolescence (i.e., Age 11 and 15)?

First, we calculated the three standard SCQ domain scores (i.e., communication, social reciprocity, and repetitive and stereotyped behaviors; Berument et al. 1999) for all SCQ items and for only QA‐characteristic items for all groups at 11 (QA, UK, & CA‐11) and 15 years (QA, UK, & CA‐15). Subsequently, we performed a one‐way ANOVA with “Group” as the independent variable at both 11 and 15 years. Post hoc testing with Bonferroni correction was performed to determine the specificity of significant group effects. Secondly, we conducted *χ* ^2^ tests with Fisher's exact test to compare the proportion of the QA group and the CA group members that displayed the QA‐characteristic items at ages 11 and 15.

#### 1.3.4 Do QA and CA Groups Show Similar Patterns of Co‐Occurring Behavioral and Emotional Difficulties During Adolescence?

We conducted one‐way ANOVA tests with group (QA vs. CA vs. UK Control) as the independent variable and ADHD, emotional and conduct problems, and IQ as the dependent variable at ages 11 (QA vs. CA‐11 vs. UK Control) and 15 years (QA vs. CA‐15 vs. UK Control). Post hoc testing was performed to determine the specificity of significant group effects.

All tests, except CFA, were run using SPSS version 26. For CFA, we used MPlus version 8.7.

## 2 Results

### 2.1 What Is the Autism Profile of the QA Group?

Adoptees within the QA group differed statistically from UK Controls on the same 19 SCQ items at both ages 11 and 15 years (Table 2), 4 communication items, 7 social reciprocity items, and 8 repetitive and stereotyped behaviors items. Out of these items, the effects for 10 persisted into adulthood: 3 from the communication domain, 2 from the social reciprocity domain, and 5 from the repetitive and stereotyped behaviors domain (Table 2). Further analyses revealed that items were equally present in males and females in the QA group (Appendix IV, Table V).

**TABLE 2:** Comparison of SCQ item endorsements between QA and UK Control groups across development.

Social communication questionnaire items		Age 11 (*n*, %)		Age 15 (*n*, %)		Age YA (*n*, %)		Group comparison (*χ* ^2^ (1)), phi		
		QA	UK	QA	UK	QA	UK	Age 11	Age 15	Age YA
Communication	Uses odd phrases	16 (61.5)	4 (8.2)	14 (63.6)	3 (6.5)	14 (70)	2 (5.3)	24.74**, 0.57	25.89**, 0.62	27.49**, 0.69
	Uses socially inappropriate questions or statements	17 (68)	8 (16.7)	18 (81.8)	3 (6.5)	14 (70)	2 (5.3)	19.23**, 0.51	39.53**, 0.76	27.49**, 0.69
	Uses madeup words	11 (42.6)	3 (6.1)	11 (52.4)	4 (8.7)	4 (21.1)	3 (7.9)	14.65**, 0.44	15.84**, 0.49	2.036, 0.19
	Gets pronouns the wrong way round	7 (26.7)	2 (4.2)	7 (31.8)	1 (2.2)	5 (25)	1 (2.6)	8.17*, 0.33	12.60**, 0.43	7.07*, 0.35
	Does not shake head to mean no	11 (44)	16 (36)	7 (33.3)	24 (54.5)	5 (25)	15 (40.5)	0.51, 0.09	2.56, −0.20	1.38, −0.16
	Does not talk to just be friendly	2 (7.7)	2 (4.2)	1 (4.5)	4 (8.7)	2 (10)	1 (2.6)	0.41, 0.07	0.38, −0.07	1.45, 0.16
	Cannot have a to and fro conversations	1 (3.8)	1 (2.1)	1 (4.5)	—	4 (21.1)	1 (2.6)	0.23, 0.06	2.12, 0.18	5.37*, 0.31
	Does not spontaneously point at things to show them	10 (38.5)	16 (34)	6 (27.3)	26 (56.5)	23 (60.5)	8 (40)	0.14, 0.04	5.11*, −0.27	2.13, −0.20
	Does not nod head to mean yes	9 (36)	15 (31.9)	7 (31.8)	24 (53.3)	5 (25)	14 (36.8)	0.12, 0.04	2.71, −0.20	0.83, −0.12
	Does not play pretend games	5 (19.2)	12 (25.5)	15 (68.2)	35 (81.4)	15 (75)	33 (86.8)	0.37, −0.07	1.43, −0.15	1.30, −0.15
Social Reciprocity	Does not show a normal range of facial expressions	8 (30.8)	—	5 (22.7)	1 (2.2)	3 (16.7)	1 (2.6)	16.88**, 0.47	7.82*, 0.34	3.63, 0.26
	Does not try to comfort parent if sad/hurt	7 (28)	1 (2.1)	7 (31.8)	1 (2.2)	3 (15)	3 (7.9)	11.57**, 0.39	12.60**, 0.43	0.71,0.11
	Does not smile back when smiled at	5 (20)	1 (2.1)	5 (23.8)	1 (2.2)	1 (5)	1 (2.6)	6.99*, 0.31	8.28*, 0.35	0.22,0.07
	Does not look directly in the face when talking	8 (32)	4 (8.3)	4 (18.2)	5 (10.9)	3 (15)	1 (2.6)	6.70*, 0.303	0.69, 0.10	3.12,0.23
	Does not have a particular or best friend	7 (26.9)	3 (6.3)	8 (36.4)	3 (6.5)	12 (60)	2 (5.3)	6.17*, 0.29	9.77**, 0.38	21.44**, 0.61
	Does not respond positively when another child approaches	5 (20)	2 (4.2)	5 (22.7)	0 (0)	4 (20)	2 (5.3)	4.75*, 0.26	11.28**, 0.41	3.068, 0.23
	Does not participate in cooperative group games	5 (20)	2 (4.2)	7 (36.8)	2 (4.4)	9 (45)	2 (5.3)	4.75*, 0.26	11.60**, 0.43	13.46**, 0.48
	Does not show appropriate facial expression to a particular situation	5 (19.2)	2 (4.2)	7 (31.8)	2 (4.3)	5 (25)	4 (10.5)	4.47*, 0.25	9.78**, 0.38	2.09, 0.19
	Does not want you to join in her enjoyment	1 (3.8)	—	2 (9.1)	3 (6.5)	2 (10)	3 (7.9)	1.87, 0.16	0.14, 0.05	0.07, 0.04
	Is not interested in children same age	6 (23.1)	6 (12.8)	9 (40.9)	13 (28.3)	13 (60)	12 (31.6)	1.30, 0.13	1.09, 0.13	4.36, 0.27
	Does not offer to share things	5 (19.2)	6 (12.2)	9 (40.9)	8 (17.4)	5 (25)	6 (15.8)	0.60, 0.09	4.40, 0.25	0.72, 0.11
	Does not play imaginative games with other	7 (26.9)	12 (26.1)	16 (80)	33 (80.5)	18 (90)	26 (68.4)	0.01, 0.01	0.01, −0.01	3.33, 0.24
	Does not use gestures with sounds or words to get attention	3 (12)	7 (14.6)	3 (13.6)	15 (33.3)	6 (30)	12 (31.6)	0.09, −0 0.04	2.92, −0.21	0.02, −0.02
	Does not show things that interest her	—	—	1 (4.5)	0 (0)	—	2 (5.3)	—	2.12, 0.18	1.09, −0.14

Social communication questionnaire items		Age 11 (*n*, %)		Age 15 (*n*, %)		Age YA (*n*, %)		Group comparison (*χ* ^2^ (1)), phi		
		QA	UK	QA	UK	QA	UK	Age 11	Age 15	Age YA
Repetitive & Stereotyped behaviors	Has odd hand or finger movements or mannerisms	10 (38.5)	—	7 (31.8)	2 (4.3)	6 (30)	2 (5.3)	21.75**, 0.54	9.78**, 0.38	6.51*, 0.34
	Is interested in part of toys	11 (42.3)	3 (6.1)	5 (23.8)	0 (0)	4 (20)	1 (2.6)	14.65**, 0.44	11.84**, 0.42	5.02*, 0.29
	Odd preoccupations	7 (26.9)	—	9 (45)	0 (0)	5 (25)	1 (2.6)	14.55**, 0.44	23.97**, 0.60	6.85*, 0.35
	Has unusual sensory interest	8 (32)	1 (2)	5 (25)	0 (0)	6 (30)	1 (2.6)	13.91**, 0.43	12.44**, 0.43	9.25**, 0.40
	Says same thing over and over	9 (34.6)	2 (4.1)	8 (38.1)	1 (2.2)	5 (25)	3 (8.3)	12.65**, 0.41	15.99**, 0.49	2.92, 0.23
	Has complicated body movements such as spinning	6 (23.1)	1 (2)	4 (18.2)	0 (0)	1 (5.6)	1 (2.8)	8.88**, 0.34	8.89**, 0.36	0.26, 0.07
	Has things that have to be done in a very particular way	7 (26.9)	2 (4.1)	5 (22.7)	2 (4.3)	5 (25)	2 (5.3)	8.39**, 0.335	5.44*, 0.28	4.81*, 0.29
	Unusually intense special interests	13 (50)	9 (18.4)	12 (54.5)	6 (13)	5 (25)	3 (7.9)	8.20**, 0.33	13.17**, 0.44	3.22, 0.24

Abbreviation: YA, young‐adulthood.

* Groups are significantly different (*p* < 0.05).

** Groups are significantly different (*p* < 0.001).

### 2.2 Do QA Group Autism Symptoms Cluster According to the Standard Autism Domains?

CFA analyses for the 19 QA‐characteristic items showed the 3‐factor model offered the best fit at both ages 11 and 15 in the group of Romanian adoptees with over 6 months of institutional deprivation (*n* = 101). The 10 QA‐characteristic adult‐persistent items also mapped onto the 3‐factor model during young adulthood, which offered the best benchmark of good fit (Table 3 and Appendix Section III for factor loadings).

**TABLE 3:** Comparison of CFA models at ages 11, 15, and young adulthood for the deprived ( >  6 months) Romanian adoptees sample.

		Chi‐square	RMSEA (90% CI)	SRMR	CFI	TLI
19 adolescent limited QA‐characteristic SCQ items	Age 11					
	3 factors	163.94*	0.03 (0.00–0.06)	0.16	0.97	0.96
	2 factors	179.88	0.05 (0.00–0.07)	0.18	0.94	0.93
	1 factor	183.9	0.05 (0.01–0.07)	0.18	0.93	0.92
	Age 15					
	3 factors	178.21*	0.04 (0.00–0.07)	0.18	0.97	0.96
	2 factors	184.31	0.05 (0.06–0.08)	0.19	0.96	0.95
	1 factor	236.79	0.08 (0.06–0.10)	0.2	0.9	0.99
10 adult persistent QA‐characteristic SCQ items	YA					
	3 factors	**39.96** *	**0.04 (0.00–0.11)**	0.12	**0.99**	**0.99**
	2 factors	**44.02** *	0.07 (0.06–0.12)	0.16	**0.98**	**0.97**
	1 factor	71.33	0.13 (0.09–0.17)	0.24	0.93	0.91

Abbreviations: CFI, Comparative Fit Index; QA, Quasi‐autism; RMSEA, Root Mean Square Error of Approximation; SCQ, Social Communication Questionnaire; SRMR, Standardized Root Mean Square Residual; TLI, Tucker‐Lewis Index; YA, young‐adulthood.

* *p* >  0.05, Figures in **bold** show figures that met the benchmark of good fit.

### 2.3 How Do Autism Symptoms in the QA and CA Groups Differ During Adolescence (i.e., Age 11 and 15)?

For the age 11 comparison, the QA and CA groups differed only on the Repetitive and Stereotyped Behaviors domain and the total score for the QA‐characteristic items. When the non‐QA SCQ items were added to the analyses, the CA‐11 group showed significantly higher scores than QA for all 3 domains and the total score. For the age 15 comparison, no differences were found between the QA and CA‐15 groups in any of the scores by domain, either for the QA‐characteristic items or the total full SCQ scale (Table 4). The variation in SCQ presentation, as represented by the standard deviation, was quite marked but was similar in magnitude for the QA and CA groups. To explore whether these patterns of differences could be accounted for by differences in general cognitive ability, we ran a series of ANCOVAs. Given that IQ was only available for a minority of the selected QUEST participants, we took a hybrid approach whereby IQ was included as a measure of general cognitive ability for the ERA sample and ABAS‐II scores were included as a proxy for IQ for the QUEST sample. ABAS‐II scores were strongly correlated with IQ in QUEST (*r* = 0.60, *p* < 0.001). This did not change the main findings *vis‐à‐vis* ERA versus QUEST differences (see Appendix IV, Tables IX and X). For the age‐11 granular analysis, the QA and CA‐11 groups differed in 3 out of the 19 QA‐characteristic items. All these were in the Repetitive and Stereotyped Behaviors domain and were more common in the CA‐11 group. However, for the remaining non‐QA items, endorsement was significantly higher in the CA‐11 group for 8 items (6 from the social reciprocity domain and 2 from the communication domain). The age 15 analysis found only 1 item from the Repetitive and Stereotyped Behaviors domain more common in the CA‐15 group. One QA‐characteristic item (i.e., uses socially inappropriate questions or statements) was significantly more common in QA than in CA‐15. Only 3 out of the 13 remaining non‐QA related items, all of them from the communication domain, were more common in the CA‐15 group (Table 5).

**TABLE 4:** Group comparison of SCQ items by domain for QA‐characteristic and full‐scale SCQ items.

Age 11	QA (*n* = 26)	CA‐11 (*n* = 21)	UK (*n* = 52)	Main effect	Post hoc test
QA‐characteristic items (mean, SD)					
Communication	1.96 (1.46)	2.05 (1.39)	0.34 (0.81)	***F* (2**,**90) = 23.95**, ***p* < 0.001**	**CA & QA** > **UK**
Social reciprocity	1.70 (2.03)	2.62 (1.56)	0.23 (0.69)	***F* (2**,**89) = 25.46**, ***p* < 0.001**	**CA & QA** > **UK**
RSB	2.72 (2.17)	4.52 (1.97)	0.37 (0.67)	***F* (2**,**92) = 60.09**, ***p* < 0.001**	**CA** > **QA** > **UK**
Total	5.90 (3.59)	9.19 (3.06)	0.95 (1.80)	***F* (2**,**89) = 76.10**, ***p* < 0.001**	**CA** > **QA** > **UK**
**All SCQ items (mean**, **SD)**	**QA (*n* = 26)**	**CA‐11 (*n* = 21)**	**UK (*n* = 52)**	**Main effect**	**Post hoc test**
Communication	3.39 (1.95)	4.90 (1.76)	1.6 (1.71)	***F* (2**,**86) = 25.99**, ***p* < 0.001**	**CA** > **QA** > **UK**
Social reciprocity	3.00 (3.22)	6.23 (3.24)	0.95 (1.41)	***F* (2**,**85) = 33.11**, ***p* < 0.001**	**CA** > **QA** > **UK**
RSB	2.72 (2.17)	4.52 (1.97)	0.37 (0.67)	***F* (2**,**92) = 60.09**, ***p* < 0.001**	**CA** > **QA** > **UK**
Total	8.75 (4.6)	16.52 (5.67)	3.12 (3.55)	***F* (2**,**80) = 64.31**, ***p* < 0.001**	**CA** > **QA** > **UK**

Age 15	QA (*n* = 21)	CA‐15 (*n* = 24)	UK (*n* = 52)	Main effect	Post hoc test
QA‐characteristic items (mean, SD)					
Communication	2.38 (1.32)	1.54 (1.47)	0.23 (0.60)	***F* (2**,**88) = 31.79**, ***p* < 0.001**	**CA & QA** > **UK**
Social reciprocity	2.21 (2.01)	2.66 (2.10)	0.22 (0.60)	***F* (2**,**85) = 25.20**, ***p* < 0.001**	**CA & QA** > **UK**
RSB	2.50 (2.12)	3.75 (2.25)	0.24 (0.43)	***F* (2**,**85) = 44.41**, ***p* < 0.001**	**CA & QA** > **UK**
Total	6.65 (3.82)	7.96 (3.74)	0.69 (1.28)	***F* (2**,**83) = 65.42**, ***p* < 0.001**	**CA & QA** > **UK**
**All SCQ items (mean**, **SD)**	**QA (*n* = 26)**	**CA‐15 (*n* = 24)**	**UK (*n* = 52)**	**Main Effect**	**Post hoc test**
Communication	3.95 (2.25)	3.96 (1.71)	2.75 (1.37)	***F* (2**,**84) = 4.85**, ***p* = 0.010**	**CA & QA** > **UK**
Social reciprocity	4.61 (3.31)	5.66 (3.69)	2.02 (1.58)	***F* (2**,**79) = 14.56**, ***p* < 0.001**	**CA & QA** > **UK**
RSB	2.5 (2.12)	3.75 (2.25)	0.24 (0.43)	***F* (2**,**92) = 44.41**, ***p* < 0.001**	**CA & QA** > **UK**
Total	10.87 (5.41)	14.29 (6.86)	5.13 (2.81)	***F* (2**,**80) = 27.35**, ***p* < 0.001**	**CA & QA** > **UK**

*Note*: Figures in **bold** show groups that are significantly different (i.e., *p* < 0.05).

Abbreviations: CA, Community Autism; QA, Quasi‐Autism; RSB, Repetitive and Stereotyped BehaviorBehaviour; SCQ, Social Communication Questionnaire; SD, Standard Deviation.

**TABLE 5:** Group comparison between QA and CA of item endorsement for QA characteristic and non‐QA‐related SCQ items.

QA‐characteristic item		Age 11 (*n*, %)		Age 15 (*n*, %)		Group comparison (*χ* ^2^ (1)), phi	
		QA (*n* = 26)	CA‐11 (*n* = 21)	QA (*n* = 26)	CA‐15 (*n* = 24)	QA vs. CA‐11	QA vs. CA‐15
Communication	Uses odd phrases	16 (61.5)	13 (61.9)	14 (63.6)	11 (45.8)	0.01, 0.01	1.47, −0.18
	Uses socially inappropriate questions or statements	17 (65.4)	13 (61.9)	18 (81.8)	11 (45.8)	0.19, −0.06	6.38*, −0.37
	Uses made‐up words	11 (42.3)	7 (33.3)	11 (52.4)	6 (25)	0.40, −0.09	3.57, −0.28
	Gets pronouns the wrong way round	7 (26.7)	10 (47.6)	7 (31.8)	9 (37.5)	2.55, 0.21	0.16, 0.06
Social reciprocity	Does not have a particular or best friend	7 (26.9)	10 (47.6)	8 (36.4)	9 (37.5)	2.55, 0.21	0.01, 0.01
	Does not participate in cooperative group games	5 (20)	9 (42.9)	7 (36.8)	7 (29.2)	2.82, 0.47	0.28, −0.08
	Does not smile back when smiled at	5 (19.2)	7 (33.3)	5 (23.8)	10 (41.7)	1.05, 0.15	1.61, 0.19
	Does not try to comfort parent if sad/hurt	7 (28)	9 (42.9)	7 (31.8)	10 (41.7)	1.11, 0.15	0.48, 0.10
	Does not show a normal range of facial expressions	8 (30.8)	6 (28.6)	5 (22.7)	9 (37.5)	0.03, −0.02	1.18, 0.16
	Does not show appropriate facial expression to a particular situation	5 (19.2)	6 (28.6)	7 (31.8)	10 (41.7)	0.56, 0.11	0.48, 0.10
	Does not respond positively when another child approaches	5 (20)	8 (38.1)	5 (22.7)	9 (37.5)	1.84, 0.20	1.18, 0.16
Repetitive & stereotyped behaviors	Has odd hand or finger movements or mannerisms	10 (38.5)	10 (47.6)	7 (31.8)	13 (54.2)	0.40, 0.09	2.33, 0.23
	Odd preoccupations	7 (26.9)	12 (57.1)	9 (45)	10 (41.7)	4.40*, 0.31	0.05, −0.03
	Has unusual sensory interest	8 (32)	11 (52.4)	5 (25)	11 (45.8)	1.96, 0.21	2.05, 0.21
	Says same thing over and over	9 (34.6)	14 (66.7)	8 (38.1)	5 (20.8)	4.78*, 0.32	1.63, −0.19
	Has unusually intense special interests	13 (50)	12 (57.1)	12 (54.5)	13 (54.2)	0 0.28, 0.07	0.01, −0.00
	Is interested in part of toys	11 (42.3)	9 (42.9)	5 (23.8)	12 (50)	0.01, 0.01	3.27, 0.27
	Has things that have to be done in a particular way	7 (26.9)	17 (81.0)	5 (22.7)	18 (75)	13.57**, 0.54	12.55**, 0.52
	Has complicated body movements	6 (23.1)	10 (47.6)	4 (18.2)	8 (33.3)	3.12, 0.26	1.37, 0.17

Non‐QA‐characteristic item		Age 11 (*n*, %)		Age 15 (*n*,%)		Group comparison (*χ* ^2^ (1)), phi	
		QA (*n* = 26)	CA‐11 (*n* = 21)	QA (*n* = 26)	CA‐15 (*n* = 24)	QA vs. CA Age 11	QA vs. CA Age 15
Communication	Does not spontaneously point at things to show them	10 (38.5)	13 (61.9)	6 (27.3)	14 (58.3)	2.55, 0.23	4.51*, 0.31
	Does not nod head to mean yes	11 (44)	9 (42.9)	7 (31.8)	7 (29.2)	0.55, −0.07	0.04, −0.04
	Does not shake head to mean no	9 (36)	11 (52.4)	7 (33.3)	7 (29.2)	0.32, 0.08	0.09, −0.05
	Does not talk to be friendly	2 (7.7)	11 (52.4)	1 (4.5)	9 (37.5)	11.60**, 0.50	7.33*, 0.40
	Cannot have a to and fro conversation	1 (4)	6 (28.6)	1 (4.5)	7 (29.2)	5.34*, 0.34	4.84*, 0.32
	Does not play pretend games	5 (19.2)	10 (47.6)	15 (68.2)	14 (58.3)	4.31, 0.30	0.48, −0.10
Social reciprocity	Does not look directly in the face when talking	8 (32)	13 (61.9)	4 (18.2)	9 (37.5)	4.11, 0.30	2.11, 0.21
	Does not show things of interest	—	5 (23.8)	1 (4.5)	6 (25.0)	6.93*, 0.38	3.72, 0.28
	Does not share things	5 (19.2)	13 (61.9)	9 (40.9)	13 (54.2)	8.95**, 0.44	0.81, 0.13
	Does not want you to join in her enjoyment	1 (3.8)	6 (28.6)	2 (9.1)	7 (29.2)	5.60*, 0.35	2.94, 0.25
	Does not use gestures with sounds or words to get attention	3 (12)	9 (42.9)	3 (13.6)	4 (16.7)	5.64*, 0.35	0.08, 0.04
	Does not play imaginative games with others	5 (19.2)	10 (47.6)	16 (80)	18 (75)	7.42**, 0.40	0.15, −0.06
	Is not interested in children same age	6 (23.1)	16 (76.2)	4 (40.9)	15 (62.5)	13.16**, 0.53	2.14, 0.22

* Groups are significantly different (*p* < 0.05).

** Groups are significantly different (*p* < 0.001).

### 2.4 Do QA and CA Groups Show Similar Patterns of Co‐Occurring Behavioral and Emotional Difficulties During Adolescence?

QA and CA groups showed similar levels of inattention and overactivity, emotional and conduct problems at both ages 11 and 15 years. These results were similar when general cognitive ability was included as a covariate (see above). Cognitive function measured by IQ levels was also similar in both groups (Table 6).

## 3 Discussion

In establishing marked autistic features as a core component of the pattern of impairment associated with severe deprivation, Rutter raised important questions about both the impact of deprivation on development and the causes of autism (Rutter et al. 1999). He also highlighted the need to understand better the clinical overlap between autism following institutional deprivation and autism as presented in non‐deprived individuals in community settings, that is, QA and CA. Our recent analysis of the QA group (Rodriguez‐Perez et al. 2023) indicated: (i) a strong continuity of autism symptoms across development in the QA group, with communication problems worsening over time; (ii) an overlap of QA group membership with the other deprivation‐specific problems seen in ERA (e.g., ADHD, DSE) and (iii) a complex set of QA‐group‐related challenges related to mental health and functional impairment in adulthood. The current study extends these analyses by providing a detailed characterization of the autism symptom profile of the QA group and then by exploring similarities and differences between the QA group and a community sample of early diagnosed autistic people. There were a number of notable findings illustrating similarities between the QA and CA but also differences.

First, with regard to similarities, the autism symptoms structure in the QA group was consistent with the three‐factor model, represented by social interaction, communication, and repetitive and stereotyped behaviors domains, outlined in the DSM‐IV (APA 2013). This suggests that autism arising following institutional deprivation and CA have a similar underlying latent structure, suggesting the equivalence of the two constructs. It is worthy of note that the current autism conceptualisation, as per the DSM‐5 (APA 2013) and ICD‐11 (Worth Health Organization 2022), refers to two domains only (social communication and restricted and repetitive behaviors). However, the SCQ was initially developed under the 3‐domain classification and did not necessarily include information about symptoms that are newly relevant in the DSM‐5 (e.g., sensory aversions). Additionally, a number of recent studies have not supported an SCQ two factor model (Hegemann et al. 2024; Uljarević et al. 2021). Some of these differences are likely due to the use of various versions of the SCQ (i.e., lifetime vs. current), as well as other factors intrinsically related to the characteristics of the sample being assessed, such as age, sex, verbal status and status (i.e., autism vs. general population).

Second, looking within‐domain, there were strong similarities in the scope of the *repetitive and stereotyped behaviors* domain between the QA and CA groups. However, while the QA‐characteristic items covered the whole RSB scale, on average, these were less often endorsed than in the CA group at age 11. In this way, our results mirror those from Wolstencroft et al. (2023), who found that repetitive and stereotyped behaviors symptoms in children with a history of early maltreatment were less common compared to non‐maltreated children with a formal autism diagnosis. Thirdly, associated emotional and behavioral problems in the QA group were prominent but no more severe than those seen in the CA group. This is in line with previous studies (i.e., Wolstencroft et al. 2023) and might ultimately suggest that the co‐occurrence of these difficulties in the QA group might not only be linked to a history of neglect but also to the autism symptoms themselves. In fact, previous studies from the Romanian adoptees have shown that the risk of adolescent emotional problems is mediated via the emergence of early neurodevelopmental problems (Golm et al. 2020).

Third, in terms of differences between the QA and CA groups, three findings stood out. First, the QA group had non‐standard communication and social reciprocity profiles. The communication profile in this group mainly involved structural language abnormalities (i.e., the use of odd‐phrases and made‐up words, using pronouns the other way round, and asking socially inappropriate questions). It is possible that the higher scores of the SCQ communication subscale in the QA group were due to them producing more language. Interestingly, Rutter previously described the QA group, in early childhood, as characterized by an unusual level of chattiness during childhood assessment (Rutter et al. 1999). This may be linked to their disinhibited style of social interaction (Kennedy et al. 2017): DSE is especially common in the QA group in adulthood (Rodriguez‐Perez et al. 2023; Sonuga‐Barke et al. 2017). Consistent with this, the item “Uses socially inappropriate questions or statements” was nearly twice as common in the QA group than in CA at age 15. This could be seen as an expression of the inappropriate over‐familiarity and failure to observe appropriate boundaries during social interaction, which is part of DSE (Kennedy et al. 2017). Second, problems with social reciprocity in the QA group during adolescence were diffuse and lacked persistence into adulthood. At least at age 11, many of the SCQ items in this domain were significantly more common in the CA group, suggesting that individuals in the QA group had a much more limited expression of difficulties related to social reciprocal interaction. Again, this could be related to the overlap of QA and DSE and needs to be addressed in future studies. Third, it appeared that symptoms were more persistent in the QA than CA group, with differences between groups in the aggregated SCQ scores by domain, either for QA‐characteristic items or the full SCQ scale of items, being only present at age 11 and disappearing by age 15. Additionally, the two groups seemed to follow different developmental trajectories. That is, in CA symptoms of autism were less marked at age 15 than 11, whereas they appeared stable in the QA group between age 11 and 15 years. In fact, our previous ERA analyses of the developmental trajectories of autism symptoms in QA from childhood to adulthood, showed a relative increase of autism symptoms in the communication domain during the transition from late childhood (i.e., age 11) to adolescence (Rodriguez‐Perez et al. 2023). Could this decrease in the CA group be due to including different participants in the analyses at age 11 and 15 in the CA groups to make these comparisons? It was possible that CA‐11 participants were simply more impaired than CA‐15, ultimately resulting in the observed decrease of autism symptoms between age 11 and 15 in this group. To further explore this, we ran a comparison of SCQ scores between CA‐11 and CA‐15 (Appendix IV, Table IV) and we found no differences between groups.

Could the high incidence of autism in the ERA sample be due to selective placement of children already at high risk for the development of autism pre‐institutionally? This seems unlikely for a number of reasons. First, in terms of elevated pre‐institutional risk, even though poverty‐related pre‐ and perinatal autism risk factors were possibly more common among families who placed their children in institutions, the small odds ratio that such risks confer, *vis‐à‐vis* autism, seen in previous studies (Mandy and Lai 2016), is way below that required to explain the massively elevated prevalence of autism in the ERA sample. Second, in terms of selective placement, the possibility that children were placed because they already had ‘autism’ even in a nascent form, seems implausible. This is because nearly all adoptees were placed in the orphanages in the first few months of life, when notwithstanding profound disability, autistic traits (of the type seen in the QA group) would have been very difficult to discern (Landa 2008; Ozonoff et al. 2009;). Finally, the selective placement for adoption hypothesis cannot account for one of the key findings from the ERA study—that autism was restricted to those individuals who experience extended exposure to institutional deprivation ( >  6 months), being, in contrast, non‐existent in the low deprivation group (< 6 months of institutional deprivation) (Rutter et al. 1999).

Why did some Romanian adoptees exposed to extended institutional deprivation develop autism? While the specific mechanisms responsible cannot be identified on the basis of the data from the ERA study, the persistence and continuity of autism into adult life, despite enrichment through adoption (Rodriguez‐Perez et al. 2023; Sonuga‐Barke et al. 2017), point to the powerful effect that exposure to extreme deprivation during early critical developmental periods can have on brain development (Rutter et al. 2004). In this regard, Rutter (Rutter et al. 2004) highlighted possible contributions of experience‐expectant and experience‐dependent brain programming mechanisms and stress‐related brain damage as three plausible mechanisms. In the former case, institutional deprivation disrupts the environmental inputs required for normal development (Nelson and Gabard‐Durnam 2020). In the second case, the deprivation‐related experiences shape the developing brain in specific ways (McLaughlin et al. 2019). In the third case, deprivation creates stress‐related hormonal responses that damage brain structure/function (Heim and Nemeroff 2001; Nemeroff 2016).

The current study had some key strengths. First, the use of common measures across deprived and non‐deprived groups to compare autism symptoms and other associated behavioral and emotional difficulties. Second, participants were matched as closely as possible on age. It also had limitations. First, the majority of SCQ‐based studies have used the *Lifetime* version (Lee et al. 2023). In the current study, we used the SCQ *Current* version, which measured symptoms over the past 3 months. However, in the ERA study, it was used at multiple ages across the lives of participants, providing a reasonable lifetime estimate. Second, the assessment of autism symptoms using the SCQ relied on a restricted range of items within each domain (see Methods section and Appendix I for rationale). This meant that we cannot rule out the fact that we might have missed important developmentally relevant characteristics in the QA group. In addition, we used this restricted range of items to run our comparison with the CA group, which might have also resulted in us missing more important relevant characteristics of the CA group. Third, since the SCQ uses binary coding, we were not able to comment on the severity of autism symptoms or assess any qualitative differences in autism symptoms between QA and CA participants (Table 5). The identification of subtle differences or degree of severity of autism symptoms would be particularly relevant in clinical practice. However, this is the first study to provide item‐level information, as previous studies of autism symptoms in children with and without a history of early maltreatment only reported aggregated symptoms by domain (Wolstencroft et al. 2023). Fourth, the majority of the QA group were females (61.5%), while in the CA group, they were mostly male (11.6% females at age 11, and 9.3% at age 15). This raises the question of whether the symptoms of autism within the QA group are driven by the sex differences in autism expression. However, there was no difference between males and females in symptom expression in either the QA or CA groups (Appendix VI, Tables V–VII). Lastly, studies have highlighted how differences in general cognitive abilities, as measured by IQ, can influence the presentation and severity of autism symptoms (Wolff et al. 2022) and the co‐occurring behavioral and emotional difficulties (Gotham et al. 2015; Kraper et al. 2017; Stringer et al. 2020). In the current study, IQ measures were only available for a minority of the selected QUEST sample. They did have a measure of adaptive functioning, the ABAS‐II (Harrison and Oakland 2003). Although not ideal, we did run a hybrid ANCOVA with ABAS‐II measures included for the QUEST sample and IQ for the ERA sample. This analysis suggested that the results obtained in the current study were not due to IQ differences (see Appendix IV, Tables IX and X).

**TABLE 6:** Group comparison of co‐occurring behavioral and emotional difficulties during adolescence.

Age 11, mean (SD)	QA (*n* = 26)	CA‐11 (*n* = 21)	UK (*n* = 52)	Main effect	Post hoc comparison
Inattention & overactivity	1.12 (1.07)	1.67 (1.15)	0.23 (0.63)	***F* (2**, **92) = 21.19**, ***p* < 0.001**	CA & QA>UK
EMO problems	0.81 (1.06)	0.86 (0.85)	0.10 (0.37)	*F* (2, 92) = 2.24, *p* = 0.112	—
Conduct problems	0.42 (0.70)	0.10 (0.30)	0.19 (0.57)	***F* (2**, **92) = 11.74**, ***p* < 0.001**	CA & QA>UK
IQ	82.20 (17.34)	68.73 (24.69)*	105.44 (15.41)	***F* (2**, **85) = 27.76**, ***p* < 0.001**	CA & QA<UK

Age 15, mean (SD)	QA (*n* = 26)	CA‐15 (*n* = 24)	UK (*n* = 45)	Main effect	Post hoc comparison
Inattention & overactivity	1.18 (1.00)	1.08 (1.00)	0.33 (0.85)	***F* (2**, **88) = 8.53**, ***p* < 0.001**	CA & QA>UK
EMO problems	0.64 (0.85)	0.21 (0.51)	0.04 (0.21)	***F* (2**, **88) = 9.95**, ***p* < 0.001**	CA & QA>UK
Conduct problems	0.41 (0.59)	0.88 (0.99)	0.11 (0.32)	*F* (2, 88) = 11.71, *p* = 0.102	—
IQ	81.75 (16.62)	65.29 (41.71)*	103.64 (15.39)	***F* (2**, **69) = 17.12**, ***p* < 0.001**	CA & QA<UK

*Note*: Figures in **bold** show groups that are significantly different (i.e., *p* < 0.05).

Abbreviations: CA, Community Autism; EMO, emotional problems; QA, Quasi‐Autism; SD, Standard Deviation.

* IQ assessment was not systematically conducted in QUEST for the whole sample. The shown figures only include 11 participants at age 11 and 6 at age 15.

There are a number of possible clinical implications of the current findings. First, the domains of social reciprocity and communication might be the most diagnostically discriminative in deprivation‐related autism, given that our study showed that individuals in the QA group displayed a much narrower coverage of autism symptoms relative to the CA group in these domains. Second, co‐occurring emotional and behavioral problems should be addressed in individuals with deprivation‐related autism to the same level they would be in individuals with autism (Pemovska et al. 2024), considering the similarities between the QA and CA groups observed in the current study. Third, in our previous analysis of developmental trajectories of autism symptoms from childhood to adulthood (Rodriguez‐Perez et al. 2023), we raised the question of whether we should continue to refer to deprivation‐related autism as QA. Given the current results highlighting the similarities in profiles between the QA and CA groups, including the similar level of associated difficulties between the two, we judge it time to drop the “*quasi*” prefix. Firstly, referring to deprivation‐related autism as 'quasi' might minimize the clinical significance of the condition and hinder an individuals' ability to access clinical services. Secondly, autism, generally, is a diagnosis based on symptoms, not cause, and we don't hesitate to use its diagnosis when other environmental factors, such as prematurity, are thought to be causative (Lord et al. 2022).

In summary, QA and CA groups share elements in common, although there are a number of specific differences. Although mapping onto the standard three‐factor model outlined in the DSM‐IV (APA 1993), the autism symptoms in the QA group show a narrower range of difficulties in the communication and social reciprocity domains relative to CA, but cannot be distinguished from CA in terms of the repetitive and stereotyped behaviors presentation. The associated level of emotional and behavioral problems in the QA group is no different from the one seen in CA and, therefore, clinical services should address these to the same level they would in any individual with autism.

## Conflicts of Interest

E.S.‐B. has received speaker fees and conference support from Medice and Takeda, research support in kind from QBTech and book royalties from OUP and Jessica Kingsley. He is the editor‐in‐chief of Journal of Child Psychology and Psychiatry for which he and his University receives financial support. T.C. has received consultancy fees from F. Hoffmann‐La Roche Ltd. and royalties from Sage Publications and Guilford Publications. E.S. receives funding from the National Institute for Health Research (IK), EU Innovative Medicines Initiative, UK Research and Innovation, King's Accelerator. She has received an honorarium from Medici. The other authors report no conflicts of interest.

## Supporting information


**Data S1.** Supporting Information.

## Data Availability

The data that support the findings of this study are available on request from the corresponding author. The data are not publicly available due to privacy or ethical restrictions.
