# Supplementary material for: What Is Distinctive About Autism Arising Following Severe Institutional Deprivation? A Direct Comparison With a Community Sample of Early Diagnosed Autistic People
Source: Autism Res. 2025 Mar 26;18(5):1062–76. doi: 10.1002/aur.70026 (PMC12123166; doi:10.1002/aur.70026)
Supplement: Supplementary file 1 — Data S1. Supporting Information. [file AUR-18-1062-s001.docx]

**Supplementary appendix**

**What is distinctive about autism arising following severe institutional deprivation? A direct comparison with a community sample of early diagnosed autistic people**

Maria Rodriguez Perez, Susie Chandler, Mark Kennedy, Tony Charman, Emily Simonoff, & Edmund Sonuga-Barke

**Contents**

I: Social Communication Questionnaire (SCQ) items excluded

II: Autism screening comparison between QA and QQA across age waves

III: Confirmatory Factor Analyses (CFA)

IV: Supplementary statistical analyses

**I: Social Communication Questionnaire (SCQ) items excluded**

**a. Items excluded in the ERA study from age 11:**

-Talks using short phrases.

-Uses your hand like a tool.

-Spontaneously copies you (or other people).

- Spontaneously join in and try to copy the actions in social games.

- Plays any pretend or make-believe games

**b. Additional items excluded from the current study only:**

- Deliberately injures her/himself.

- Has particular objects that has to carry around.

- Looks up and pays attention when you come into a room and start talking.

*Note*. Item description from the SCQ is withheld due to copyright.

**II: Autism screening comparison between QA and QQA across age waves**

**Table I: Autism Screening comparison between QA and QQA adoptees at age 6, 11, 15 and Young Adulthood**

|  | **6 years** | |  | **11 years** | | |  |
| --- | --- | --- | --- | --- | --- | --- | --- |
|  | QA (n=15) | QQA (n=11) | QA *vs* QQA | QA (n=15) | QQA (n=11) | | QA *vs* QQA |
| **Autism Screening - SCQ, mean (SD)** |  |  |  |  |  |  |  |
| Social Reciprocal Interaction | 0.93 (1.38) | 0.09 (.30) | **t= 2.28, p= .04** | 1.58 (1.65) | 0.63 (1.20) | | t= 1.61, p= .12 |
| Communication | 2.14 (1.17) | 2.35 (1.50) | t= -0.41, p= .68 | 1.73 (1.01) | 2.45 (1.75) | | t= -1.22, p= .24 |
| Repetitive & Stereotyped behaviours | 2.05 (1.31) | 1.30 (1.49) | t= 1.33, p= .20 | 1.70 (.99) | 1.63 (1.96) | | t= 0.10, p= .92 |
| SCQ Total Score | 5.01 (2.48) | 4.02 (2.04) | t= 1.04, p= .31 | 5.03 (2.27) | 4.73 (3.80) | | t= 0.25, p= .80 |
|  | **15 years** | |  |  | **YA** |  |  |
|  | QA (n=12) | QQA (n=10) | QA *vs* QQA | QA (n=11) | | QQA (n=9) | QA *vs* QQA |
| **Autism Screening - SCQ, mean (SD)** |  |  |  |  |  |  |  |
| Social Reciprocal Interaction | 2.00 (1.95) | 0.50 (.85) | **t= 2.25, p= .03** | 1.11 (1.37) | | 0.44 (.73) | t= 1.33, p= .21 |
| Communication | 2.27 (1.40) | 2.40 (1.26) | t= -0.23, p= .83 | 2.27 (1.25) | | 1.89 (1.61) | t= 0.50, p= .60 |
| Repetitive & Stereotyped behaviours | 1.95 (1.77) | 1.11 (1.45) | t= 1.15, p= .27 | 1.64 (1.74) | | 0.89 (1.69) | t= 0.97, p= .35 |
| SCQ Total Score | 6.11 (3.57) | 3.99 (2.48) | t= 0.17, p= .13 | 5.04 (3.67) | | 3.22 (1.98) | t= 1.33, p= .20 |

*Note*. QA, adoptees identified with QA symptoms during childhood assessment. Childhood QQA, adoptees identified with queried QA symptoms during childhood assessment.

SCQ, Social Communication Questionnaire. SD, Standard Deviation. YA, Young adult (average age 23 years).

Figures in **bold** refer to significant effect (i.e., p < .05).

**III: Confirmatory Factor Analyses (CFA)**

**Table II: Confirmatory Factor Analyses (CFA) items split**

| 3-factor model | | 2-factor model | | Single-factor model | |
| --- | --- | --- | --- | --- | --- |
| **Communication** | Uses odd phrases | **Socio-Communication** | Uses odd phrases | **ASD single factor** | Uses odd phrases |
|  | Uses socially inappropriate questions or statements |  | Uses socially inappropriate questions or statements |  | Uses socially inappropriate questions or statements |
|  | Uses made-up words |  | Uses made-up words |  | Uses made-up words |
|  | Gets pronouns the wrong way round |  | Gets pronouns the wrong way round |  | Gets pronouns the wrong way round |
| **Social reciprocity** | Does not have a particular or best friend |  | Does not have a particular or best friend |  | Does not have a particular or best friend |
|  | Does not participate in cooperative group games |  | Does not participate in cooperative group games |  | Does not participate in cooperative group games |
|  | Does not smile back when smiled at |  | Does not smile back when smiled at |  | Does not smile back when smiled at |
|  | Does not try to comfort parent if sad/hurt |  | Does not try to comfort parent if sad/hurt |  | Does not try to comfort parent if sad/hurt |
|  | Does not show a normal range of facial expressions |  | Does not show a normal range of facial expressions |  | Does not show a normal range of facial expressions |
|  | Does not show appropriate facial expression to a particular situation |  | Does not show appropriate facial expression to a particular situation |  | Does not show appropriate facial expression to a particular situation |
|  | Does not respond positively when another child approaches |  | Does not respond positively when another child approaches |  | Does not respond positively when another child approaches |
| **Repetitive & Stereotyped behaviours** | Has odd hand or finger movements or mannerisms | **Repetitive & Stereotyped behaviours** | Has odd hand or finger movements or mannerisms |  | Has odd hand or finger movements or mannerisms |
|  | Odd preoccupations |  | Odd preoccupations |  | Odd preoccupations |
|  | Has unusual sensory interest |  | Has unusual sensory interest |  | Has unusual sensory interest |
|  | Says same thing over and over |  | Says same thing over and over |  | Says same thing over and over |
|  | Has unusually intense special interests |  | Has unusually intense special interests |  | Has unusually intense special interests |
|  | Is interested in part of toys |  | Is interested in part of toys |  | Is interested in part of toys |
|  | Has things that have to be done in a particular way |  | Has things that have to be done in a particular way |  | Has things that have to be done in a particular way |
|  | Has complicated body movements |  | Has complicated body movements |  | Has complicated body movements |

*Note*. Item description from the SCQ is withheld due to copyright.

**CFA Age 11 – 3-Factor Model loadings**

| Two-Tailed | | |
| --- | --- | --- |
| Estimate S.E. Est./S.E. P-Value | | |
| SRI BY |  |  |
| S23 0.942 0.055 17.079 0.000 | | |
| S21 0.596 0.134 4.437 0.000 | | |
| S17 0.888 0.091 9.777 0.000 | | |
| S32 0.513 0.144 3.566 0.000 | | |
| S28 0.853 0.104 8.233 0.000 | | |
| S31 0.792 0.117 6.761 0.000 | | |
| S24 0.940 0.069 13.688 0.000 | | |
|  |  |  |
| COM BY |  |  |
| S9 0.895 0.079 11.391 0.000 | | |
| S11 0.788 0.088 8.976 0.000 | | |
| S12 0.767 0.102 7.531 0.000 | | |
| S13 0.711 0.112 6.327 0.000 | | |
|  |  |  |
| RSB BY |  |  |
| S38 0.919 0.078 11.752 0.000 | | |
| S34 0.484 0.125 3.880 0.000 | | |
| S33 0.634 0.124 5.098 0.000 | | |
| S36 0.710 0.113 6.312 0.000 | | |
| S14 0.876 0.087 10.089 0.000 | | |
| S39 0.609 0.163 3.733 0.000 | | |
| S35 0.549 0.135 4.057 0.000 | | |
| S30 0.577 0.125 4.596 0.000 | | |

**CFA Age 15 – 3-Factor Model loadings**

| Two-Tailed | | |
| --- | --- | --- |
| Estimate S.E. Est./S.E. P-Value | | |
|  |  |  |
| SRI BY |  |  |
| S23 0.801 0.088 9.091 0.000 | | |
| S21 0.691 0.118 5.839 0.000 | | |
| S17 0.894 0.070 12.844 0.000 | | |
| S32 0.780 0.116 6.715 0.000 | | |
| S28 0.882 0.069 12.807 0.000 | | |
| S31 0.589 0.153 3.837 0.000 | | |
| S24 1.032 0.040 26.026 0.000 | | |
|  |  |  |
| COM BY |  |  |
| S9 0.917 0.057 16.134 0.000 | | |
| S11 0.829 0.075 11.129 0.000 | | |
| S12 0.821 0.099 8.273 0.000 | | |
| S13 0.948 0.067 14.175 0.000 | | |
|  |  |  |
| RSB BY |  |  |
| S38 0.803 0.099 8.135 0.000 | | |
| S34 0.600 0.140 4.293 0.000 | | |
| S33 0.971 0.043 22.333 0.000 | | |
| S36 0.938 0.073 12.850 0.000 | | |
| S14 0.873 0.094 9.274 0.000 | | |
| S39 0.850 0.096 8.851 0.000 | | |
| S35 0.706 0.118 5.970 0.000 | | |
| S30 0.763 0.095 8.003 0.000 | | |
|  | | |

**CFA Age Young Adulthood – 3-Factor Model loadings**

Two-Tailed

Estimate S.E. Est./S.E. P-Value

SRI BY

S31 0.862 0.095 9.068 0.000

S32 0.877 0.098 8.992 0.000

COM BY

S9 0.977 0.076 12.817 0.000

S11 0.873 0.079 10.999 0.000

S12 0.485 0.184 2.630 0.009

S13 0.488 0.211 2.317 0.020

RSB BY

S14 0.810 0.102 7.920 0.000

S33 1.016 0.017 59.711 0.000

S36 0.919 0.054 17.149 0.000

S38 0.826 0.090 9.158 0.000

**Table III: Item description**

| Communication | Social Reciprocity | RSB |
| --- | --- | --- |
| S9. odd phrases | S17. smiles back when smiled at | S30. unusual special interests |
| S11. socially inappropriate questions | S21. tries to comfort | S33. odd preoccupations |
| S12. gets pronouns wrong | S23. normal range of facial expressions | S34. interest in part of toys |
| S13. made up words | S24. appropriate facial expression | S35. ritualised behaviours |
|  | S28. responds positively to others | S36. unusual sensory interests |
|  | S31. cooperative group games | S38. odd mannerisms/movements |
|  | S32. special or best friends | S39. stereotyped behaviours |

*Note*. Item description from the SCQ is withheld due to copyright.

**IV: Supplementary analyses**

**Table IV: Autism symptoms group comparison between those included and excluded from the QUEST sample**

|  | **CA Excluded (n = 160)** | **CA-11 (n = 21)** | **CA-15 (n = 24)** | **Main effect** |
| --- | --- | --- | --- | --- |
| **Communication (Mean, SD)** | 4.38 (2.07) | 5 (1.87) | 3.96 (2.26) | F (2,204) = 1.44, p = .24 |
| **Social Reciprocity (Mean, SD)** | 5.60 (3.15) | 6.24 (3.24) | 5.67 (3.69) | F (2,204) = 0.36, p = .70 |
| **Repetitive and Stereotyped behaviours (Mean, SD)** | 4.26 (2.33) | 4.52 (1.97) | 3.75 (2.25) | F (2,204) = 0.71, p = .49 |
| **SCQ Total score (Mean, SD)** | 15.45 (6.39) | 16.62 (5.79) | 14.29 (6.86) | F (2,204) = 0.75, p = .48 |

CA, Community autism. SD, Standard deviation. SCQ, Social Communication Questionnaire

**Table V: Comparison of autism symptoms by domain for QA-characteristic and all SCQ items between CA-11 and CA-15**

| **QA-characteristic items (mean, SD)** | **CA-11 (n=21)** | **CA-15 (n=24)** | **CA-11 *vs*. CA-15** |
| --- | --- | --- | --- |
| **Communication** | 2.05 (1.40) | 1.54 (1.47) | t = 1.18, p = .25 |
| **Social Reciprocity** | 2.62 (1.56) | 2.67 (2.10) | t = -0.08, p = .93 |
| **RSB** | 4.52 (1.97) | 3.75 (2.25) | t = 1.22, p = .12 |
| **Total** | 9.19 (3.06) | 7.96 (3.74) | t = 1.20, p = .12 |
| **All SCQ items (mean, SD)** | **QA (n=26)** | **CA (n=21)** | **CA-11 *vs*. CA-15** |
| **Communication** | 4.90 (1.76) | 3.96 (2.26) | t = 0.13, p = .13 |
| **Social Reciprocity** | 6.24 (3.24) | 5.67 (3.96) | t = 0.55, p = .29 |
| **RSB** | 4.52 (1.97) | 3.75 (2.25) | t = 1.22, p = .12 |
| **Total** | 16.52 (5.67) | 14.29 (6.86) | t = 1.18, p = .25 |

SCQ, Social Communication Questionnaire. SD, Standard Deviation. RSB, Repetitive and Stereotyped Behaviours. CA-11, community autism group aged 11. CA-15, community autism group aged 15.

**Table VI: Sex analysis of QA-characteristic SCQ items for QA group (age 11)**

| **SCQ Item** | | **Females (n, %)** | | | **Males (n, %)** | | **Group comparison** |
| --- | --- | --- | --- | --- | --- | --- | --- |
|  |  | **Yes** | | **No** | **Yes** | **No** |  |
| **Communication** | Uses odd phrases | | 9 (56.3) | 7 (43.8) | 7 (70) | 3 (30) | X^2^ (1) = 0.49, p = .68, phi = .48 |
|  | Uses socially inappropriate questions | | 10 (66.7) | 5 (33.3) | 7 (70) | 3 (30) | X^2^ (1) = 0.03, p = 1.00, phi = .86 |
|  | Gets pronouns wrong | | 6 (37.5) | 10 (62.5) | 1 (10) | 9 (90) | X^2^ (1) = 2.37, p = .190, phi = .12 |
|  | Uses made up words | | 8 (50) | 8 (50) | 3 (30) | 7 (50) | X^2^ (1) = 1.08, p = .428, phi = .32 |
| **Social reciprocity** | Does not have a particular or best friend | | 4 (25) | 12 (75) | 3 (30) | 7 (70) | X^2^ (1) = 0.08, p = 1.0, phi = -.06 |
|  | Does not participate in cooperative group games | | 3 (18.8) | 13 (81.3) | 2 (22.2) | 7 (77.8) | X^2^ (1) = 0.04, p = 1.0, phi = -.042 |
|  | Does not smile back when smiled at | | 5 (31.3) | 11 (68.8) | 0 (0) | 9 (100) | X^2^ (1) = 3.52, p = .12, phi = .06 |
|  | Does not try to comfort parent if sad/hurt | | 4 (26.7) | 11 (73.3) | 3 (30) | 7 (70) | X^2^ (1) = 0.03, p = 1.00, phi =.-.04 |
|  | Does not show a normal range of facial expression | | 4 (25) | 12 (75) | 4 (40) | 6 (60) | X^2^ (1) = 0.65, p = .67, phi = -.16 |
|  | Does not show appropriate facial expression to a particular situation | | 2 (12.5) | 14 (87.5) | 3 (30) | 7 (70) | X^2^ (1) = 1.21, p = .34, phi = -.22 |
|  | Does not respond positively to others | | 3 (18.8) | 13 (81.3) | 2 (22.2) | 7 (77.8) | X^2^ (1) = 0.04, p = 1.00, phi = -.04 |
| **Repetitive and stereotyped behaviours** | Has odd hand or finger movements or mannerisms | | 5 (31.3) | 11 (68.8) | 5 (50) | 5 (50) | X^2^ (1) = 0.91, p = .43, phi = -.19 |
|  | Has odd preoccupations | | 4 (25) | 12 (75) | 3 (30) | 7 (30) | X2 (1) = 0.08, p = 1.00, phi = -.06 |
|  | Has unusual sensory interests | | 4 (25) | 12 (75) | 4 (44.4) | 5 (55.6) | X^2^ (1) = 1.00, p = .39, phi = -.20 |
|  | Says same thing over and over | | 7 (43.8) | 9 (56.3) | 2 (20) | 8 (80) | X^2^ (1) = 1.53, p = .40, phi = .24 |
|  | Is interested in part of toys | | 4 (25) | 12 (75) | 7 (70) | 3 (30) | X^2^ (1) = 5.11, p = .06, phi = -.44 |
|  | Has things that need to be done in a particular way | | 4 (25) | 12 (75) | 4 (44.4) | 5 (55.6) | X^2^ (1) = 1.00, p = .39, phi = -.20 |
|  | Has unusually intense special interests | | 8 (50.0) | 8 (50) | 5 (50) | 5 (50) | X^2^ (1) = 0.00, p = 1.00, phi = .00 |
|  | Has complicated body movements | | 2 (12.5) | 14 (87.5) | 4 (40) | 6 (60) | X^2^ (1) = 2.62, p = .16, phi = -.32 |

“Yes” refers to item endorsement. SCQ, Social Communication Questionnaire.

**Table VII: Sex analysis of QA-characteristic SCQ items for CA group (age 11)**

| **SCQ Item** | | **Females (n, %)** | | **Males (n, %)** | | **Group comparison** |
| --- | --- | --- | --- | --- | --- | --- |
|  |  | **Yes** | **No** | **Yes** | **No** |  |
| **Communication** | Uses odd phrases | 3 (60) | 2 (40) | 10 (62.5) | 6 (37.5) | X^2^ (1) = 0.01, p = 1.00, phi = -.02 |
|  | Uses socially inappropriate questions | 3 (60) | 2 (40) | 10 (62.5) | 6 (37.5) | X^2^ (1) = 0.01, p = 1.00, phi = -.02 |
|  | Gets pronouns wrong | 2 (40) | 3 (60) | 8 (50) | 8 (50) | X^2^ (1) = 0.15, p = 1.00, phi =-.09 |
|  | Uses made up words | 1 (20) | 4 (80) | 6 (37.5) | 10 (62.5) | X^2^ (1) = 0.53, p = .62, phi = -.16 |
| **Social reciprocity** | Does not have a particular or best friend | 3 (60) | 2 (40) | 8 (50) | 8 (50) | X^2^ (1) = 0.15, p = 1.00, phi = .09 |
|  | Does not participate in cooperative group games | 1 (20) | 4 (80) | 8 (50) | 8 (50) | X^2^ (1) = 1.40, p = .34, phi = -.26 |
|  | Does not smile back when smiled at | 1 (20) | 4 (80) | 6 (37.5) | 10 (62.5) | X^2^ (1) = 0.53, p = .62, phi = -.16 |
|  | Does not try to comfort parent if sad/hurt | 2 (40) | 3 (60) | 7 (43.8) | 9 (56.3) | X^2^ (1) = 0.02, p = 1.00, phi = -.03 |
|  | Does not show a normal range of facial expression | 2 (40) | 3 (60) | 4 (25) | 12 (75) | X^2^ (1) = 0.42, p = .59, phi = .14 |
|  | Does not show appropriate facial expression to a particular situation | 0 (0) | 5 (100) | 6 (37.5) | 10 (62.5) | X^2^ (1) = 2.63, p = .26, phi = -.35 |
|  | Does not respond positively to others | 3 (60) | 2 (40) | 5 (31.3) | 11 (68.8) | X^2^ (1) = 1.34, p = .33, phi = .25 |
| **Repetitive and stereotyped behaviours** | Has odd hand or finger movements or mannerisms | 3 (60) | 2 (40) | 7 (43.8) | 9 (56.2) | X^2^ (1) = 0.40, p = .64, phi = .14 |
|  | Has odd preoccupations | 2 (40) | 3 (60) | 10 (62.5) | 6 (37.5) | X^2^ (1) = 0.79, p = .61, phi = -.19 |
|  | Has unusual sensory interests | 3 (60) | 2 (40) | 8 (50) | 8 (50) | X^2^ (1) = 0.15, p = 1.00, phi = .09 |
|  | Says same thing over and over | 1 (20) | 4 (80) | 10 (62.5) | 6 (37.5) | X^2^ (1) = 0.53, p = .62, phi = .16 |
|  | Is interested in part of toys | 4 (80) | 1 (20) | 8 (50) | 8 (50) | X^2^ (1) = 1.40, p = .34, phi = -.26 |
|  | Has things that need to be done in a particular way | 4 (80) | 1 (20) | 13 (81.2) | 3 (18.8) | X^2^ (1) = 0.00, p = 1.00, phi = -.01 |
|  | Has unusually intense special interests | 2 (40) | 3 (60) | 10 (62.5) | 6 (37.5) | X^2^ (1) = 0.79, p = .61, phi = -.19 |
|  | Has complicated body movements | 3 (60) | 2 (40) | 7 (43.8) | 9 (56.3) | X^2^ (1) = 0.40, p = .64, phi = .14 |

“Yes” refers to item endorsement. SCQ, Social Communication Questionnaire.

**Table VIII: Sex analysis of QA-characteristic SCQ items for CA group (age 15)**

| **SCQ Item** | | **Females (n, %)** | | **Males (n, %)** | | **Group comparison** |
| --- | --- | --- | --- | --- | --- | --- |
|  |  | **Yes** | **No** | **Yes** | **No** |  |
| **Communication** | Uses odd phrases | 2 (50) | 2 (50) | 9 (45) | 11 (55) | X^2^ (1) = 0.03, p = .86, phi = .04 |
|  | Uses socially inappropriate questions | 2 (50) | 2 (50) | 9 (45) | 11 (55) | X^2^ (1) = 0.03, p = .86, phi = .04 |
|  | Gets pronouns wrong | 2 (50) | 2 (50) | 13 (65) | 7 (35) | X^2^ (1) = 0.32, p =..62, phi =.12 |
|  | Uses made up words | 1 (25) | 3 (75) | 5 (25) | 15 (75) | X^2^ (1) = 1.00, p = 1.00, phi = .00 |
| **Social reciprocity** | Does not have a particular or best friend | 1 (25) | 3 (75) | 8 (40) | 12 (60) | X^2^ (1) = 0.32, p = 1.00, phi = -.16 |
|  | Does not participate in cooperative group games | 2 (50) | 2 (50) | 5 (25) | 15 (75) | X^2^ (1) = 1.00, p = .55, phi = .21 |
|  | Does not smile back when smiled at | 2 (50) | 2 (50) | 8 (40) | 12 (60) | X^2^ (1) = 0.14, p = .1.00, phi = .08 |
|  | Does not try to comfort parent if sad/hurt | 2 (50) | 2 (50) | 8 (40) | 12 (60) | X^2^ (1) = 0.14, p = .1.00, phi = .08 |
|  | Does not show a normal range of facial expression | 2 (50) | 2 (50) | 7 (35) | 13 (65) | X^2^ (1) = 0.32, p = .62, phi = .12 |
|  | Does not show appropriate facial expression to a particular situation | 0 (0) | 4 (100) | 10 (50) | 10 (50) | X^2^ (1) = 3.43, p = .11, phi = -.38 |
|  | Does not respond positively to others | 3 (75) | 1 (25) | 6 (30) | 14 (70) | X^2^ (1) = 2.88, p = .13, phi = .35 |
| **Repetitive and stereotyped behaviours** | Has odd hand or finger movements or mannerisms | 3 (75) | 1 (25) | 10 (50) | 10 (50) | X^2^ (1) = 0.84, p = .60, phi = .19 |
|  | Has odd preoccupations | 2 (50) | 2 (50) | 8 (40) | 12 (60) | X^2^ (1) = 0.14, p = 1.00, phi = .08 |
|  | Has unusual sensory interests | 1 (25) | 3 (75) | 8 (40) | 12 (60) | X^2^ (1) = 1.65, p = .30, phi = .26 |
|  | Says same thing over and over | 1 (25) | 3 (75) | 4 (20) | 16 (80) | X^2^ (1) = 1.53, p = .40, phi = .24 |
|  | Is interested in part of toys | 4 (100) | 0 (0) | 8 (40) | 12 (60) | X^2^ (1) = 4.80, p = .09, phi = .45 |
|  | Has things that need to be done in a particular way | 3 (75) | 1 (25) | 15 (75) | 5 (25) | X^2^ (1) = 1.00, p = 1.00, phi = .00 |
|  | Has unusually intense special interests | 2 (50) | 2 (50) | 11 (55) | 9 (45) | X^2^ (1) = 0.03, p = 1.00, phi = -.04 |
|  | Has complicated body movements | 2 (50) | 2 (50) | 6 (30) | 14 (70) | X^2^ (1) = 0.60, p = .58, phi = .16 |

“Yes” refers to item endorsement. SCQ, Social Communication Questionnaire.

**Table IX: Group comparison of SCQ items by domain for QA-characteristic and full scale SCQ items covarying for IQ* levels**

| **Age 11** |  |  |  |  |  |  |
| --- | --- | --- | --- | --- | --- | --- |
| **QA-characteristic items (mean, SD)** | **QA (n=26)** | **CA-11 (n=21)** | **UK (n=52)** | **Overall Model** | **Between-subjects effect (Group)** | **Group comparison** |
| **Communication** | 1.96 (1.46) | 2.05 (1.39) | 0.34 (0.81) | **F (3,88) = 16.9,7 p < .001,** $\boldsymbol{R}^{\boldsymbol{2}}$**= .35** | **F (2, 88) = 20.31, p < .001** | **CA & QA > UK** |
| **Social Reciprocity** | 1.70 (2.03) | 2.62 (1.56) | 0.23 (0.69) | **F (3,87) = 28.16, p < .001,** $\boldsymbol{R}^{\boldsymbol{2}}$ **= .36** | **F (2, 87) = 13.07, p < .001** | **CA & QA > UK** |
| **RSB** | 2.72 (2.17) | 4.45 (1.98) | 0.37 (0.67) | **F (3, 90) = 39.45, p < .001,** $\boldsymbol{R}^{\boldsymbol{2}}$ **= .55** | **F (2, 90) = 37.02, p < .001** | **CA > QA > UK** |
| **Total** | 5.90 (3.59) | 9.19 (3.06) | 0.95 (1.80) | **F (3, 84) =. 51.26, p < .001,** $\boldsymbol{R}^{\boldsymbol{2}}$ **= .63** | **F (2, 84) = 48.27, p < .001** | **CA > QA > UK** |
| **All SCQ items (mean, SD)** | **QA (n=26)** | **CA-11 (n=21)** | **UK (n=52)** | **Overall Model** | **Between-subjects effect (Group)** | **Group comparison** |
| **Communication** | 3.39 (1.95) | 4.90 (1.76) | 1.6 (1.71) | **F (3,84) = 19.550, p < .001,** $\boldsymbol{R}^{\boldsymbol{2}}$**= .39** | **F (2, 84) = 13.27, p < .001** | **CA & QA > UK** |
| **Social Reciprocity** | 3.00 (3.22) | 6.23 (3.24) | 0.95 (1.41) | **F (3,83) = 26.13, p < .001,** $\boldsymbol{R}^{\boldsymbol{2}}$ **= .47** | **F (2, 83) = 9.67, p < .001** | **CA > QA & UK** |
| **RSB** | 2.72 (2.17) | 4.52 (1.97) | 0.37 (0.67) | **F (3, 90) = 39.45, p < .001,** $\boldsymbol{R}^{\boldsymbol{2}}$**= .55** | **F (2, 90) = 37.02, p < .001** | **CA > QA > UK** |
| **Total** | 8.75 (4.6) | 16.52 (5.67) | 3.12 (3.55) | **F (3, 79) = 54.83, p < .001,** $\boldsymbol{R}^{\boldsymbol{2}}$ **= .66** | **F (2, 79) = 26.27, p < .001** | **CA > QA > UK** |
| **Age 15** |  |  |  |  |  |  |
| **QA-characteristic items (mean, SD)** | **QA (n=26)** | **CA-15 (n=24)** | **UK (n=52)** | **Overall Model** | **Between-subjects effect (Group)** | **Group comparison** |
| **Communication** | 2.38 (1.32) | 1.54 (1.47) | 0.23 (0.60) | **F (3,75) = 15.62, p < .001,** $\boldsymbol{R}^{\boldsymbol{2}}$ **= .36** | **F (2, 75) = 17.28, p < .001** | **CA & QA > UK** |
| **Social Reciprocity** | 2.21 (2.01) | 2.66 (2.10) | 0.22 (0.60) | **F (3,77) = 21.26, p < .001,** $\boldsymbol{R}^{\boldsymbol{2}}$ **= .44** | F (2, 77) = 2.74, p = .07 | **-** |
| **RSB** | 2.50 (2.12) | 3.75 (2.25) | 0.24 (0.43) | **F (3,74) = 29.06, p < .001,** $\boldsymbol{R}^{\boldsymbol{2}}$ **= .52** | **F (2,74) = 9.63, p < .001** | **CA & QA > UK** |
| **Total** | 6.65 (3.82) | 7.96 (3.74) | 0.69 (1.28) | **F (3,73) = 46.79, p < .001,** $\boldsymbol{R}^{\boldsymbol{2}}$ **= .64** | **F (2, 73) = 16.26, p < .001** | **CA & QA > UK** |
| **All SCQ items (mean, SD)** | **QA (n=26)** | **CA-15 (n=24)** | **UK (n=52)** | **Overall Model** | **Between-subjects effect (Group)** | **Group comparison** |
| **Communication** | 3.95 (2.25) | 3.96 (1.71) | 2.75 (1.37) | **F (3,72) = 3.81, p = .014,** $\boldsymbol{R}^{\boldsymbol{2}}$ **= .10** | F (2, 72) = 0.91, p = .41 | **-** |
| **Social Reciprocity** | 4.61 (3.31) | 5.66 (3.69) | 2.02 (1.58) | **F (3,68) = 15.33, p < .001,** $\boldsymbol{R}^{\boldsymbol{2}}$ **= .38** | F (2, 68) = 0.58, p = .56 | **-** |
| **RSB** | 2.5 (2.12) | 3.75 (2.25) | 0.24 (0.43) | **F (3,74) = 29.06, p < .001,** $\boldsymbol{R}^{\boldsymbol{2}}$ **= .52** | **F (2,74) = 9.63, p < .001** | **CA & QA > UK** |
| **Total** | 10.87 (5.41) | 14.29 (6.86) | 5.13 (2.81) | **F (3,68) = 26.71, p < .001,** $\boldsymbol{R}^{\boldsymbol{2}}$ **= .52** | F (2, 68) = 1.76, p =.18 | **-** |

SD, Standard Deviation. RSB, Repetitive and Stereotyped Behaviours.

Figures in **bold** show groups that are significantly different (i.e., p < 0.05).

SCQ,Social Communication Questionnaire. RSB, Repetitive and Stereotyped Behaviour. QA, Quasi-Autism. CA, Community Autism

*The Adaptive Behaviour Assessment System (ABAS-II; Harrison and Oakland, 2003) was used as a measure of cognitive functioning for IQ in the QUEST sample only.

**Table X: Group comparison of co-occurring behavioural and emotional difficulties during adolescence covarying for IQ* levels**

| **Age 11, Mean (SD)** | **QA (n = 26)** | **CA-11 (n = 21)** | **UK (n = 52)** | **Main Effect** | **Between-subjects effect (Group)** | **Group comparison** |
| --- | --- | --- | --- | --- | --- | --- |
| **Inattention & Overactivity** | 1.12 (1.07) | 1.67 (1.15) | 0.23 (0.63) | **F (3, 88) = 16.04, p < .001,** $\boldsymbol{R}^{\boldsymbol{2}}$ **= .33** | **F (2,88) = 5.25, p < .001** | **CA & QA > UK** |
| **EMO Problems** | 0.81 (1.06) | 0.86 (0.85) | 0.11 (0.37) | **F (3, 88) = 9.40, p < .001,** $\boldsymbol{R}^{\boldsymbol{2}}$ **= .22** | **F (2,88) = 6.90, p = .002** | **CA & QA > UK** |
| **Conduct Problems** | 0.42 (0.70) | 0.10 (0.30) | 0.19 (0.57) | **F (3, 88) = 2.39, p = .094,** $\boldsymbol{R}^{\boldsymbol{2}}$ **= .04** | F (2,88) = 3.064, p = .054 | - |
| **Age 15, Mean (SD)** | **QA (n = 26)** | **CA-15 (n = 24)** | **UK (n = 45)** | **Main Effect** | **Between-subjects effect (Group)** | **Group comparison** |
| **Inattention & Overactivity** | 1.18 (1.00) | 1.08 (1.00) | 0.33 (0.85) | **F (3, 75) = 3.90, p = .012,** $\boldsymbol{R}^{\boldsymbol{2}}$ **= .10** | F (2, 75) = 1.56, p = .218 | - |
| **EMO Problems** | 0.64 (0.85) | 0.21 (0.51) | 0.04 (0.21) | **F (3, 75) = 5.28, p = .002,** $\boldsymbol{R}^{\boldsymbol{2}}$ **= .14** | **F (2, 75) = 5.52, p = .006** | **CA & QA > UK** |
| **Conduct Problems** | 0.41 (0.59) | 0.88 (0.99) | 0.11 (0.32) | **F (3, 75) = 6.38, p < 0.001,** $\boldsymbol{R}^{\boldsymbol{2}}$ **= .35** | **F (2, 75) = 4.812, p = .041** | **CA > UK** |

Figures in **bold** show groups that are significantly different (i.e., p < 0.05).

EMO, emotional problems. SD, Standard Deviation. QA, Quasi-Autism. CA, Community Autism.

*The Adaptive Behaviour Assessment System (ABAS-II; Harrison and Oakland, 2003) was used as a measure of cognitive functioning for IQ in the QUEST sample only.

**Note for Tables IX and X**: Unfortunately, although IQ was available for all ERA participants, it was available for only a minority of selected QUEST participants (11 out of 21 eleven-year-olds and 6 out of 24 fifteen-year-olds) at those ages. This precluded a meaningful assessment of the impact of adding IQ as a covariate. The QUEST participants did, however, have a measure of adaptive functioning which could be considered a proxy for IQ (i.e., Adaptive Behaviour Assessment System (ABAS-II) (Harrison & Oakland, 2003). This was strongly correlated with IQ (r = .60, p < .001). We therefore ran a series of ANCOVA’s tests including hybrid covariate – IQ in ERA and ABAS-III in QUEST. The results align with the primary findings in the manuscript, with CA and QA displaying differences in domains at age 11, for both QA-characteristic items and the entire range of SCQ items, which subside by age 15. Similarly, the pattern of results CA *vs* QA in terms of co-occurring behavioural and emotional difficulties is consistent with the main analysis, with both groups showing similar levels of inattention and overactivity, emotional and conduct problems at both age 11 and 15 years.
